# Gonadotropin-secreting and thyrotropin-secreting pituitary adenomas: A single-center experience

**DOI:** 10.20945/2359-4292-2023-0072

**Published:** 2023-12-01

**Authors:** Manjiri Karlekar, Chakra Diwaker, Vijaya Sarathi, Anurag Lila, Anima Sharma, Saba Samad Memon, Virendra Patil, Tushar Bandgar

**Affiliations:** 1 Seth G.S. Medical College and KEM Hospital Department of Endocrinology Mumbai India Department of Endocrinology, Seth G.S. Medical College and KEM Hospital, Mumbai, India; 2 Vydehi Institute of Medical Sciences and Research Centre Department of Endocrinology Bangalore India Department of Endocrinology, Vydehi Institute of Medical Sciences and Research Centre, Bangalore, India

**Keywords:** Functional gonadotropinoma, TSHoma, thyrotropinoma, gonadotropinoma

## Abstract

**Objective:**

Data regarding rare FPAs from India, a resource limited setting, are limited. We describe a case series of rare FPAs from a single center in western India.

**Materials and methods:**

This was a retrospective case record review of patients diagnosed between January 2010 and July 2022. The diagnosis was based on biochemical (inappropriately elevated serum FSH/LH) and pathologic (positive immunostaining for FSH/LH) features in patients with FGA, and elevated serum thyroid hormones and normal/elevated TSH in patients with TSHomas.

**Results:**

We identified 11 patients with a total of six FGAs (median age 43.5 years, five men, one FGA cosecreting TSH, median largest dimension 40 mm, range 33–60 mm) and six TSHomas (median age 34.5 years, four women, two TSHomas cosecreting GH, median largest dimension 42.5 mm, range 13–60 mm). Symptoms of sellar mass effects led to pituitary imaging in most patients with FGA. Patients with TSHomas had symptoms of excess hormone secretion (GH/TSH) or sellar mass effects. The TSHomas that cosecreted GH/FSH were larger than those secreting only TSH. Transsphenoidal resection was the most common first-line therapy but significant residual disease was frequent (3 out of 6 FGAs and 4 out of 5 TSHomas).

**Conclusion:**

This is the first and second case series of FGAs and TSHomas, respectively, from India. In this study, TSHomas presented at younger age, were larger and had low surgical cure rates.

## INTRODUCTION

The incidence and prevalence of pituitary adenomas are approximately 4/year/100,000 and 115/100,000 individuals, respectively (1,2). Notably, a large proportion of pituitary adenomas are nonsecretory and are labeled as nonfunctioning pituitary adenomas (NFPAs). Among functioning pituitary adenomas (FPAs), prolactinomas are the most common types, followed by adenomas that secrete growth hormone (GH) and adrenocorticotrophic hormone (ACTH). Pituitary adenomas that secrete thyroid-stimulating hormone (TSH) and gonadotropins are rare, comprising only 1%-2% of all FPAs (1). Among NFPAs with positive immunostaining for gonadotropins, only a fraction secrete gonadotropins into circulation and are labeled as functioning gonadotroph adenomas (FGAs). Most FGAs in men are clinically silent or “whispering” adenomas, and very few have clinical manifestations related to gonadotropin excess (3). In addition to clinical symptoms related to sellar mass effect, other specific clinical manifestations of FGAs due to autonomous secretion of follicle-stimulating hormone (FSH) are precocious puberty in children, menstrual irregularities and/or ovarian hyperstimulation syndrome in premenopausal women, and testicular enlargement in men. Autonomous luteinizing hormone (LH) secretion may manifest as androgen excess. Given the rarity of FGAs, systematic series on their optimal management are lacking. The usual line of management is surgical resection of the adenoma followed by external radiotherapy for the residual lesion (4).

Other rare FPAs are TSH-secreting pituitary adenomas (TSHomas), which present with varying degrees of hyperthyroidism with or without sellar mass effects (5). Historically, these tumors were often misdiagnosed as Graves’ disease, but with newer TSH assays, the biochemical differentiation between both has become easy (6). Resistance to thyroid hormone is another differential diagnosis of TSHomas; the presence of a macroadenoma on magnetic resonance imaging (MRI) and the absence of germline *TRH*β mutations favor the diagnosis of TSHoma (7). The primary therapy for TSHomas is surgical excision after control of hyperthyroidism with medical treatment directed to either the thyroid (thionamides) or the pituitary adenoma (somatostatin analogues). Residual disease after surgery is managed with long-acting somatostatin analogues and/or pituitary-directed radiotherapy (7).

We describe herein our experience in managing FGAs and TSHomas at a tertiary care center in western India.

## SUBJECTS AND METHODS

This study was conducted after approval from the institutional ethics committee. A waiver of consent was granted, given the study's retrospective nature. Case records of pituitary adenomas managed at a tertiary health center in western India from January 2010 to July 2022 were reviewed. Six patients with FGA were identified, and one of the FGAs cosecreted TSH. Additionally, five patients with TSHomas were identified, and two of the TSHomas cosecreted GH. Detailed history including demographic features, clinical characteristics (presenting features and associated complaints), serum hormonal levels (FSH, LH, testosterone, estradiol, prolactin, total/free triiodothyronine [TT3/FT3], total/free thyroxine [TT4/FT4], TSH, GH, insulin-like growth factor 1 [IGF1], and cortisol), radiological (pituitary MRI), treatment, and outcome details were recorded.

The diagnosis of FGA was based on biochemical (elevated FSH/LH or nonsuppressed FSH/LH in the setting of elevated sex steroid) and pathological confirmation of FGA (positive immunostaining for FSH/LH), with or without clinical features of gonadotropin excess (macrotestes/hyperandrogenism in men, and multicystic ovaries/ovarian hyperstimulation syndrome/oligomenorrhea in women) (8). The diagnosis of TSHoma was based on elevated thyroid hormone levels (TT3/FT3 or TT4/FT4) in the presence of normal or increased TSH levels. Pituitary MRI was performed using a 1.5-Tesla scanner, T1- and T2-weighted images, sagittal and coronal sections, and gadolinium contrast enhancement. Levels of TT3, TT4, FT3, FT4, TSH, FSH, LH, testosterone, estradiol, cortisol, GH, and prolactin were measured using chemiluminescence assay on a LIAISON analyzer (DiaSorin, Saluggia, Italy) with an intra-assay and interassay coefficient of variation (CV) of < 10%.

## RESULTS

### Functional gonadotroph adenomas (FGAs)

**Figure 1:**
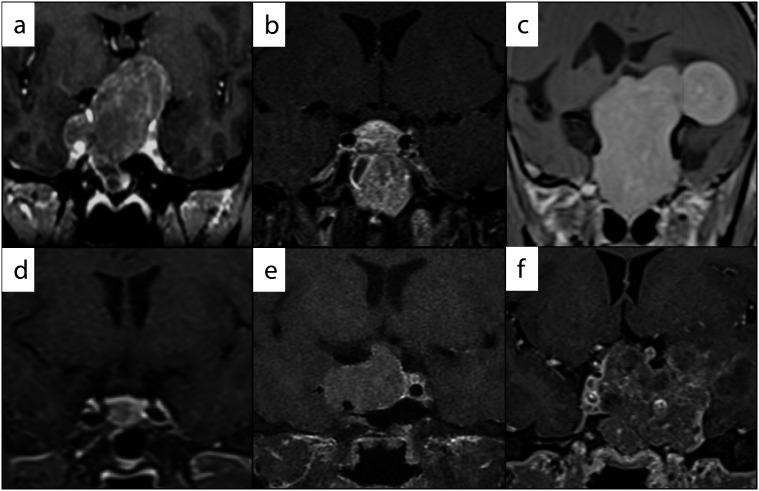
Preoperative contrast-enhanced MRI images of pituitary adenomas in patients (P) with gonadotropinomas and TSHomas. (a) P1: 30 × 23 × 42 mm with suprasellar extension; (b) P2: 34 × 28 × 29 mm, with predominant infrasellar extension; (c) P4: 42 × 37 × 60 mm, giant tumor extending into the left temporal lobe, compressing the lateral ventricles; (d) P7: 10 × 11 × 13 mm, tumor confined to the sella; (e) P8: 32 × 26 × 20 mm, predominant right parasellar extension; (f) P11: 44 × 60 × 40 mm, heterogenous tumor extending into the left temporal lobe and infrasellar extension.

Six patients (five men) with FGA were identified (Table 1). All tumors secreted FSH, while two tumors (in two men, *i.e.*, P2 and P3) cosecreted LH (identified on the basis of elevated serum total testosterone, *i.e.*, > 1,000 ng/dL), and one tumor (in P6) cosecreted TSH. The median age at FGA diagnosis was 43.5 years (range 25-55 years). All patients (except for P6) presented with chronic symptoms of sellar mass effects like headache (P2, P5), decreased vision in the form of blurred vision or peripheral visual field defects (P1, P3, P4), and recent-onset seizures (P4), leading to imaging of the brain and diagnosis of pituitary macroadenoma (Figure 1). Notably, P6 had thyrotoxic symptoms for 4 years and was on variable dosages of oral carbimazole. He was diagnosed incidentally with pituitary macroadenoma when a psychiatrist ordered a brain MRI to evaluate pathological gambling and compulsive buying that the patient had for a year.

**Table 1:** Clinical and biochemical characteristics of patients with functional gonadotroph adenomas

Patient	Age years)/sex	Presenting feature (duration in months)	Adenoma dimensions on MRI (mm)	Serum biochemistry*						Management	Pituitary adenoma staining on IHC
				FSH (mIU/mL)	LH (mIU/mL)	Testosterone (ng/dL)	Prolactin (ng/mL)	Central hypothyroidism	Central hypercortisolism		
P1	25/F	Oligomenorrhea (24), decreased vision (8.0)	30 × 23 × 42	18.3	2.3	N/A	382	No	No	TSS, RT, OCP	FSH (+), LH (−), prolactin (−)
P2	51/M	Headache (2.0)	34 × 28 × 29	208	>250	>1,500	15.2	No	No	TSS, RT	FSH (+), LH (+)
P3	42/M	Decreased vision (3.0)	38 × 25 × 26	31.5	5.25	1,259	37.4	Yes	No	TSS	FSH (+), LH (+)
P4	55/M	Decreased vision (108), seizure (1.0)	42 × 37 × 60	400	22.2	319	34.47	No	No	TSS	FSH (+), LH (−)
P5	45/M	Headache (2.0)	33 × 29 × 25	34.26	5.06	18	21	Yes	No	TSS	FSH (+) (LH N/A)
P6	42/M	Thyrotoxic symptoms (48), impulse-control disorder (12)	55 × 38 × 42	42.89	8.36	952	27	No*	No	TSS twice, RT, octreotide	FSH (+), LH (−), TSH (+)

M, male; F, female; MRI, magnetic resonance imaging; FSH, follicle-stimulating hormone; LH, luteinizing hormone; IHC, immunohistochemistry; N/A, not available; OCP, combined oral contraceptive pills; RT, radiotherapy; TSS, transsphenoidal surgery; TSH, thyroid-stimulating hormone. *Normal reference values of serum hormone levels: FSH (men and women in follicular phase of menstrual cycle), 2.5-10 mIU/mL; LH (men and women in follicular phase of menstrual cycle), 2.5-10 mIU/mL; testosterone (men), 300-1,100 ng/dL; prolactin, 5-25 ng/mL.

The median FSH was 38.5 mIU/mL (range 18.3-400 mIU/mL). Bilateral testicular size, measured using a Prader orchidometer, was ≥ 25 mL in 3 of 5 male patients (P3, P5, and P6) and 20 mL in the remaining 2 male patients (P2 and P4). Male patients (P2 and P3) with elevated serum total testosterone (>1,000 ng/dL) had no specific symptoms of hyperandrogenism, like hypersexuality or priapism. In contrast, P1 had a history (1.5 years before FGA diagnosis) of oligomenorrhea, multicystic ovaries (135 mL and 129 mL) with increased endometrial thickness (17.3 mm), and serum prolactin > 200 ng/mL. She was started on oral cabergoline 0.5 mg per week, which reduced her prolactin level to 0.5 ng/mL after a month, when the cabergoline was then stopped. Her serum prolactin (measured in 1:10 dilution) was 382 ng/mL before transsphenoidal surgery (TSS) and declined to 14.5 ng/mL 3 months after the procedure. After TSS, she had a residual parasellar lesion. At that time, stimulation with GnRH analogue (subcutaneous triptorelin 100 µg) increased the serum FSH (from 12.5 mIU/mL to 30 mIU/mL) and LH (from 2.8 mIU/mL to 17.4 mIU/mL) from baseline to 4 hours respectively. This patient received fractionated radiotherapy for the residual parasellar tumor and cyclical estrogen and progesterone replacement for the persistent amenorrhea. A reassessment 3 years after radiotherapy (after stopping cyclical estrogen and progesterone for 1 month), basal serum FSH was 5.37 mIU/mL and estradiol was 10 pg/mL.

All five male patients were managed with TSS, which was followed by radiotherapy in two of them (P2 and P6) due to tumor progression (P2) and persistent hyperthyroidism (P6). At the latest follow-up after TSS and radiotherapy (18 months after diagnosis), P2 had serum levels of LH, FSH, and testosterone of 56.8 mIU/mL, 12.44 mIU/mL, and 996 ng/dL, respectively. At the latest follow-up, 24 months after TSS, P3 had panhypopituitarism and required replacement with cortisol, androgen, and thyroid hormone. For P4 and P5, follow-up details were not available. Follow-up details of P6 are described below.

### TSH-secreting pituitary adenomas (TSHomas)

Six patients (four women) with TSHomas (of which two cosecreted GH [P10 and P11] and one cosecreted FSH [P6]) were identified, and their median age at diagnosis was 34.5 years (range 21-42 years) (Table 2). Four patients presented with symptoms of sellar mass effects, like chronic headache (P7, P8, and P9) (Figure 1) and acute headache with a seizure episode (P10), leading to brain MRI scanning and diagnosis of pituitary macroadenoma. A later biochemical evaluation revealed secondary hyperthyroidism. On inquiry, P7 reported having thyrotoxic symptoms for 5 years, which had not been evaluated. P10 presented with coarse facial features and acral enlargement; her biochemistry showed elevated serum IGF1 (647.5 ng/mL) and GH (23.6 ng/mL). P11 presented with secondary amenorrhea, acral enlargement, diabetes, and a large goiter, and the diagnosis of acromegaly was suspected. Biochemistry showed elevated serum IGF1 (1,372.5 ng/mL) and secondary hyperthyroidism (Figure 1). The median TSH level of the patients with TSHomas was 4.81 µIU/mL (range 0.88-17.94 µIU/mL).

**Table 2:** Clinical and biochemical characteristics of patients with TSH-secreting pituitary adenomas (TSHomas)

Patient	Age (years)/sex	Presenting feature (duration in months)	Adenoma dimensions on MRI (mm)	Biochemistry					Preoperative management (duration in days)	Management	Pituitary adenoma staining on IHC
				TSH (0.4-4 µIU/mL)	T3 (81-178 ng/dL)	FT3 (2.3-6.6 pg/mL)	T4 (4.5-11.5 µg/dL)	FT4 (0.8-1.8 ng/dL)			
P6*	42/M	Tremors and restlessness (48), impulse control disorder (12)	55 × 38 × 42	17.94	270	14.9#	12.23	3.79#	†N/A	TSS (twice), RT, octreotide	FSH (+), LH (−) TSH (+)
P7	40/F	Anxiety and palpitation (60), headache (18)	10 × 11 × 13	9.79	318	7.04	21.4	2.39	Carbimazole tablet 30 mg BD (10), SSKI 5 drops QID (10), octreotide 100 µg s.c. TDS (10)	TSS	N/A
P8	29/M	Headache (10), erectile dysfunction (10)	32 × 26 × 20	4.27	249	6.2	14.6	2.01	Carbimazole tablet 10 mg BD (5)	TSS, RT	TSH diffuse +, GH patchy + PRL patchy +
P9	21/F	Headache (48), visual field defect (12)	47 × 40 × 25	5.36	164	N/A	12.75	N/A	Carbimazole tablet 30 mg BD (15)	TSS, planned for RT	TSH diffuse +
P10@	42/F	Acute headache, episode of seizure, acral enlargement	38 × 38 × 32	3.05	N/A	6.05	N/A	3.74	Octreotide 100 µg s.c. TDS (5)	TSS, planned for repeat TSS	TSH diffuse + GH patchy ve+
P11@	29/F	Amenorrhea (120), acral enlargement (96), diabetes (6)	44 × 60 × 40	0.88	243	7.22	18.2	3.21	Carbimazole tablet 30 mg BD (30), SSKI 5 drops QID (30), octreotide 100 µg, s.c. TDS (30)	Total thyroidectomy	N/A

M, male; F, female; MRI, magnetic resonance imaging; IHC, immunohistochemistry; TSH, thyroid-stimulating hormone; T3/FT3, total and free triiodothyronine; T4/FT4, total and free thyroxine; N/A, not available; TSS, transsphenoidal surgery; RT, radiotherapy; FSH, follicle-stimulating hormone; LH, luteinizing hormone; GH, growth hormone; PRL, prolactin; SSKI, saturated solution of potassium iodide; s.c., subcutaneous; BD, twice a day; QID, four times a day; TDS, three times a day.

† Details of preoperative management are not available.

* P6 had a pituitary adenoma cosecreting FSH and TSH (also mentioned in Table 1).

# Blood for measurement of FT3 and FT4 values was collected after starting treatment with carbimazole, and the values were not measured concomitantly to the baseline T3, T4, and TSH mentioned in the table.

@ P10 and P11 had adenomas cosecreting TSH and GH; these patients had GH levels of 23.6 ng/mL and 25 ng/mL, respectively, and IGF1 levels of 647.5 ng/mL and 1,372 ng/mL, respectively.

The patients’ presurgical medical management with carbimazole, saturated solution of potassium iodide, and/or octreotide is detailed in Table 2. All patients with TSHomas (except for P11) were initially managed with TSS (twice in P6), which was followed by radiotherapy in two patients (P6 and P8) due to residual tumor with persistent hyperthyroidism. After TSS and radiotherapy, P6 had increased serum TSH (16.2 µIU/mL) and FSH (94.7 mIU/mL) levels, for which monthly octreotide long-acting release (LAR) depot (20 mg intramuscular) was initiated. This treatment reduced hormonal secretions (TSH to 4.62 µIU/mL and FSH to 18.2 mIU/mL), but the patient stopped it after 6 months. At the latest follow-up (9 years), he was asymptomatic and euthyroid. P7 achieved remission after TSS and was euthyroid on follow-up evaluation (2 years). P8 was euthyroid with normal gonadal axis (after TSS, serum total testosterone increased from 60 ng/dL to 446 ng/dL) 1 year after radiotherapy. At the time of publication, radiotherapy for functioning residual TSHoma was planned for P9. P10 had a residual tumor secreting GH and TSH that was amenable to debulking 1 year after TSS; she was advised to undergo a repeat TSS but has not followed up at our center since then. P11 underwent total thyroidectomy for resection of a goiter with retrosternal extension and compressive features; pituitary adenomectomy has been planned for her.

## DISCUSSION

We described herein 11 adults with rare FPAs, of whom 5 had tumors secreting more than one hormone. In our series, FGAs and TSHomas were equally prevalent. Notably, FGAs had a male predominance and presented exclusively with sellar mass effects. In contrast, TSHomas presented a decade earlier than FGAs, had a female predominance, and manifested in the majority of the cases with sellar mass effects or features of acromegaly. All patients had macroadenomas. Surgical resection and radiotherapy were the most common first- and second-line treatment modalities.

The age of the patients at FGA presentation in our study (median 43.5 years, range 25-55 years) is comparable to that reported in previous series by Beckers and cols. (36-57 years), Snyder and cols. (median 56 years, range 32-75 years), and Cote and cols. (median 52 years, range 32-71 years) (8–10). A male predominance has been consistently observed in patients with FGA from unselected large pituitary adenoma cohorts, as noted in our cohort (8,10). Male predominance has also been observed in silent gonadotroph adenomas, which contrasts with the female predominance observed in null-cell adenomas (11). However, the reasons for male predominance in FGAs are not clear. One reason could be the underdiagnosis of FGA in older women since the distinction between FGAs and NFPAs is difficult in postmenopausal women, in whom serum concentrations of FSH and LH are elevated (12).

Men with FGA usually present with symptoms of sellar mass effects, as also noted in our study (8). Testicular enlargement (length > 6 cm) has been reported in 6 of 23 male patients with FGA (10). Notably, three men with FGA in our study had testicular size above the population average, although they had not perceived any change in testicular size (13). Two men with supranormal testosterone levels had no symptoms related to hyperandrogenism. Hence, careful clinical examination (testicular size) and biochemistry may aid in suspecting the FGA diagnosis. Unlike men, women with FGAs present more often (86.7%) with menstrual disorders, whereas headache (23.1%) and visual deficits (26.2%) are less frequent (14). However, pituitary tumor was not suspected in the only woman (P1) with FGA in our study, despite oligomenorrhea for 1.5 years, until she developed decreased vision. Interestingly, this patient had markedly enlarged multicystic ovaries (a feature in most premenopausal women with FGA) that did not receive due consideration. Hence, a high index of suspicion is necessary to diagnose FGA in both men and women.

In premenopausal women with menstrual disturbances and enlarged multicystic ovaries, FGA must be differentiated from other conditions such as primary hypothyroidism or, rarely, partial estrogen synthetic defects (aromatase deficiency, P450 oxidoreductase deficiency, or 17-alpha-hydroxylase deficiency) or partial estrogen resistance, especially during adolescence or young adulthood for the latter conditions. Our patient also had endometrial hyperplasia, which suggests effective hyperestrogenemia. Endometrial hyperplasia with hyperestrogenemia may occur in primary hypothyroidism, but the latter can be easily diagnosed with thyroid function testing. Increased serum FSH and estrogen levels are features of estrogen resistance but are not features of endometrial hyperplasia, whereas estrogen synthesis defects have FSH elevation but lack both hyperestrogenemia and endometrial hyperplasia.

Notably, the diagnosis of FGA was not suspected at initial presentation in P1, and a serum prolactin level > 200 ng/mL in this patient was treated empirically with cabergoline. Hyperprolactinemia has been described in approximately 80% of women with FGA, but levels > 150 ng/mL are rare (about 8% of the cases) (14). Hyperprolactinemia in FGA could be due to the stalk effect, prolactin cosecretion, or hyperestrogenemia. Prolactin cosecretion in FGAs is very rare, and the negative immunostaining for prolactin in our patient refuted this possibility, whereas hyperprolactinemia > 200 ng/mL is unusual due to stalk compression alone. Hyperprolactinemia of up to 334 ng/mL has been reported in girls with estrogen-secreting juvenile granulosa cell tumors and has been attributed to marked hyperestrogenemia (15). Similarly, hyperestrogenemia due to spontaneous ovarian hyperstimulation syndrome might have contributed to marked hyperprolactinemia in our patient. Notably, a response to cabergoline (serum prolactin < 5 ng/mL), as seen in our patient, may not help differentiate the causes of hyperprolactinemia as this may be a feature of all the aforementioned causes of hyperprolactinemia.

In clinical practice, FGA closely mimics and is often misdiagnosed as NFPA. Most FGAs, both in men and women, secrete FSH. In men, FGAs manifest primarily as elevated serum gonadotropins (10). Hence, an elevated FSH in men with an apparent NFPA should prompt a diagnosis of FGA. In contrast, although FSH was elevated in the female patient in our study, most women with FGA have inappropriately normal FSH (14,16). Hence, an additional search for elevated serum estradiol and/or prolactin, suppressed/inappropriately normal LH, and/or enlarged multicystic ovaries may be sought to increase the diagnostic possibilities of FGA among women with apparent NFPA.

Notably, FGAs cosecreting LH or secreting exclusively LH are rare, whereas clinical manifestations attributable to LH excess (erythrocytosis or hypersexuality) are even rarer (17–19). In FGAs secreting only FSH, LH and testosterone levels are usually low or normal, whereas in FGAs cosecreting LH, the classical hormonal profile is the elevation of both serum testosterone and LH levels. P2 had such a classical hormonal profile suggestive of LH secretion. Interestingly, two men with FGA having elevated testosterone but inappropriately normal LH – similar to P3 in our study – have been recently reported in Pakistan (20). Notably, P6 also had a profile similar to P3; however, in this patient with TSH cosecretion, a slightly elevated total testosterone may be the effect of an increase in sex hormone-binding globulin due to hyperthyroidism rather than LH cosecretion, as LH immunostaining was negative.

Besides LH, TSH is the most commonly cosecreted hormone in FSH-secreting adenomas, but only a few cases of FGAs cosecreting TSH have been reported to date (Table 3) (21–25). P6 is the one additional such case that presented in the fifth decade with a giant pituitary adenoma. In contrast, FGAs cosecreting TSH in reported cases are relatively smaller in size and present at a younger age (median 34 years) (Table 3). The smaller size may be due to earlier diagnosis due to thyrotoxic symptoms, whereas the younger age may reflect the early age at the development of pluripotent tumors.

**Table 3:** Clinical and biochemical characteristics of patients with FSH and TSH cosecreting adenomas

Authors, year	Age (years)/sex	Presenting feature	Adenoma dimensions on radiological imaging (mm)	Serum biochemistry							Management	Pituitary adenoma staining on IHC
				TSH (µIU/mL)	T3 (ng/dL)	FT3 (pg/mL)	T4 (µg/dL)	FT4 (ng/dL)	FSH (mIU/mL)	LH (mIU/mL)		
Koide and cols., 1982	23/M	Visual field defect		32*	137		10.3		63	23.7	Open adenoma resection (twice), RT (twice)	FSHβ +, FSH -ve, LH -ve, TSH -ve
Bermingham and cols., 1989	34/M	Excessive sweating, heat intolerance, palpitations	(CT) pituitary adenoma with 16 mm	11.1	316		16.3		23 (0-20)	17 (up to 25)	TSS	N/A
Sy and cols., 1992	59/M	Palpitations, heat intolerance, loss of libido, right side heart failure	(MRI) 19 mm	19			19.96		35	47 (normal testosterone)	Daily octreotide 100 µg s.c. TDS	N/A
Patrick and cols., 1994	44/M	Palpitations, weight loss		17.3	3.02	8.2		8.56	7.9 U/L	2.1 U/L	TSS	TSH+, FSH+, GH+
Vargas and cols., 2017	19/F	Headache, history of precocious puberty at the age of 7 years, (bilateral oophorectomy for ovarian cysts)	(MRI) 20×14×13	2.6				1.9	59 (post-oophorectomy)	36 (post-oophorectomy)	TSS	FSHβ+, TSHβ+
Karlekar and cols. (current study), 2023	42/M	Tremors and restlessness, impulse control disorder	(MRI) 55×38×42	17.94	270	N/A	12.23	N/A	42.89	8.36	TSS (twice), RT	TSH,+ FSH+

* TSHoma diagnosed on the basis of incomplete suppression of TSH by T3. Abbreviations: M, male; F, female; MRI, magnetic resonance imaging; CT, computed tomography; TSH, thyroid-stimulating hormone; T3/FT3, total and free triiodothyronine; T4/FT4, total and free thyroxine; FSH, follicle-stimulating hormone; LH, luteinizing hormone; IHC, immunohistochemistry; TSS, transsphenoidal surgery; RT, radiotherapy; GH, growth hormone; N/A, not available.

As also noted in our study, FGAs are almost universally macroadenomas. The first-line treatment for these rare functioning tumors is TSS. All our patients underwent TSS. Serum FSH and estradiol levels decrease sharply after surgical resection of FGA in premenopausal women. A successful surgery also usually resolves symptoms of ovarian hyperstimulation syndrome or precocious puberty in most patients. However, the female patient with FGA in our cohort had residual tumor postoperatively with persistent hormonal excess. In a recent literature review by Wang and cols. (14), around 17% of premenopausal women with FGA required second-line therapy (radiotherapy or repeat surgery), whereas in a series by Cote and cols. (8), second-line therapy was required in three of seven patients with FGA. Similarly, 3 of our 6 patients received radiotherapy as second-line therapy. Long-term outcome data on FGA are scarce. Tumor recurrence has been reported to be significantly lower in silent gonadotropinomas than null-cell adenomas (11). Studies with long follow-up are warranted to identify the long-term outcomes in patients with FGA.

Another rare subgroup of FPAs is that of TSHomas. Notably, these tumors have been identified more often in the past few years due to better awareness and availability of sensitive TSH assays (26). A recent structured review reported that the mean age at diagnosis of patients with TSHomas is 45-46 years and that these tumors have a slight female predominance (51.6%) (5). In a case series (n > 5), the mean age at diagnosis ranged from 35 to 56 years, and the proportion of men varied from 22% to 83% (27). The proportion of men was relatively lower (33%, 2 out of 6) in our cohort, whereas the average age at diagnosis was approximately 34 years, which suggests that our cohort was relatively younger. Three quarters of the patients with TSHomas present with features of thyrotoxicosis, while one quarter of them have visual field defects (5). Most of our patients were evaluated for symptoms of sellar mass effect or clinical acromegaly, with subsequent diagnosis of TSHoma after identification of a pituitary adenoma on imaging. Indeed, P10 had symptoms of thyrotoxicosis for 4 years before presenting with acromegalic features.

Interestingly, patients with TSHomas who present with thyrotoxic features are often misdiagnosed as having primary hyperthyroidism and treated with antithyroid drugs or radioactive iodine ablation. This practice may lead to an inadvertent increase in tumor size, a corollary to Nelson's syndrome (6). Hence, patients with normal/elevated TSH and elevated FT4 should not be started on antithyroid treatment in haste. A close differential diagnosis of this hormonal profile is resistance to thyroid hormone. Distinguishing the two conditions may require measurement of the alpha subunit of TSH or various dynamic tests like thyrotropin-releasing hormone stimulation, T3 suppression, or octreotide suppression (7). Fortunately, only one of our patients had received long-term therapy directed to the thyroid, and none of the patients required special tests to investigate resistance to thyroid hormone, since pituitary macroadenoma was evident on imaging studies.

The most common cosecreting pituitary adenomas are TSHomas (37.9%), and their most common cosecreted hormone is GH (57.5%), followed by prolactin (41.4%), FSH (26.5%), and LH (8.8%) (5). The more frequent cosecretion of GH and prolactin in TSHomas is due to their common origin in the PIT1 lineage. Aligned with that, half of the patients with TSHomas in our series had cosecreting tumors, most commonly GH.

Unlike FGAs, which are universally macroadenomas, only 80% of the TSHomas are macroadenomas. The mean diameter of TSHomas is approximately 20 mm (5). In our cohort, the TSHomas were larger (mean maximum tumor dimension of 40.8 ± 17.13 mm), which may be due to the frequent cosecreting tumors. In our series, cosecreting TSHomas were relatively larger (38 mm, 55 mm, and 60 mm in the largest dimension) than TSHomas secreting only TSH. According to a report, macro-TSHomas are more frequently plurihormonal in nature than micro-TSHomas (51.1% *versus* 27.3%, respectively) (5). This may be due to a greater proliferative potential of tumor cells originating from relatively immature pluripotent cells.

The first line of treatment for TSHomas is surgical resection. Euthyroid or near euthyroid status preoperatively is desired and is usually attained with a short course of antithyroid drugs and/or somatostatin analogues, as done in our cohort (7). All our patients with TSHomas underwent TSS as first-line treatment, except for P11, who was awaiting surgery at publication time. An undetectable TSH level within 7 days after surgery is considered a good predictor of surgical cure and restoration of normal response to dynamic tests (7). In our cohort, P7 was the only patient with an undetectable TSH level after TSS; this patient did not require further treatment, whereas all other patients required second-line treatment (radiotherapy or somatostatin analogues). In a recent structured review, 25% of the patients had some residual disease, whereas 16%-29% and 8%-17% received after surgery somatostatin analogues and radiotherapy, respectively (5). The higher rate of residual disease and need for second-line therapy by the patients with TSHomas in our cohort may be due to larger tumors with frequent parasellar extension. Notably, another Indian study has also reported a lower remission rate (28).

Our study is limited by the small sample size and retrospective design with its inherent limitations. Other limitations are missing immunohistochemistry data in some TSHomas and short follow-up. However, this is the first and second case series of FGAs and TSHomas, respectively, reported from India, a setting with limited resources and unique challenges in managing these rare tumors.

In conclusion, FGAs and TSHomas are rare tumors and require increased suspicion for their diagnoses. As in previous FGA cohorts, our patients with FGAs were predominantly male and presented mostly with sellar mass effects. The patients with TSHomas in our cohort had frequent cosecretory tumors, as reported in previous cohorts, but were younger, had larger tumors, and progressed with high rates of residual disease after surgery, frequently requiring second-line therapy. Larger studies are warranted to characterize better these rare FPAs in Asian-Indian patients.
